# SABERTOOTH: protein structural alignment based on a vectorial structure representation

**DOI:** 10.1186/1471-2105-8-425

**Published:** 2007-10-31

**Authors:** Florian Teichert, Ugo Bastolla, Markus Porto

**Affiliations:** 1 Institut für Festkörperphysik, Technische Universität Darmstadt, Hochschulstr. 6-8, 64289 Darmstadt, Germany; 2 Centro de Biología Molecular "Severo Ochoa", (CSIC-UAM), Cantoblanco, 28049 Madrid, Spain

## Abstract

**Background:**

The task of computing highly accurate structural alignments of proteins in very short computation time is still challenging. This is partly due to the complexity of protein structures. Therefore, instead of manipulating coordinates directly, matrices of inter-atomic distances, sets of vectors between protein backbone atoms, and other reduced representations are used. These decrease the effort of comparing large sets of coordinates, but protein structural alignment still remains computationally expensive.

**Results:**

We represent the topology of a protein structure through a structural profile that expresses the global effective connectivity of each residue. We have shown recently that this representation allows explicitly expressing the relationship between protein structure and protein sequence. Based on this very condensed vectorial representation, we develop a structural alignment framework that recognizes structural similarities with accuracy comparable to established alignment tools. Furthermore, our algorithm has favourable scaling of computation time with chain length. Since the algorithm is independent of the details of the structural representation, our framework can be applied to sequence-to-sequence and sequence-to-structure comparison within the same setup, and it is therefore more general than other existing tools.

**Conclusion**

We show that protein comparison based on a vectorial representation of protein structure performs comparably to established algorithms based on coordinates. The conceptually new approach presented in this publication might assist to unify the view on protein comparison by unifying structure and sequence descriptions in this context. The framework discussed here is implemented in the 'SABERTOOTH' alignment server, freely accessible at .

## Background

Comparing protein structures is a major issue in structural and evolutionary biology. Structure comparison allows discovering evolutionary and functional relationships that are beyond the reach of methods based only on sequences. There are two major demands for an alignment algorithm: First, it has to recognize small but significant structural similarities very efficiently and it has to produce accurate alignments even for far relatives, in order to get maximum profit from available data. Second, it must perform its computation in very short time. In fact, when all-vs-all comparison of all structures in a database is needed, a very large amount of pairwise alignments is required. For the Protein Data Bank (PDB [1]) all-vs-all alignment would take around 160 years of computation time for a tool that needs one second for a single alignment (about ≈ 10^5 ^protein chains known, July 2007).

If comparisons are not carried out accurately enough, evolutionary and functional information gets shrouded in background noise. If comparisons are too slow, some large scale analyses could become unfeasible. The above stipulates requirements for an alignment framework that go beyond currently available tools.

Here we propose a novel alignment framework that allows dealing with structure-to-structure, structure-to-sequence and sequence-to-sequence alignments in the same scheme, exploiting a vectorial representation of both sequences and structures [2]. On the one hand, these vectorial representations of protein structures reduce three-dimensional atom coordinate data to just one real number per amino acid [3], allowing fast comparisons. On the other hand, as shown in [2,4], the structural profiles used are deeply related to sequence representations like the ones discussed in [2,5,6] that are naturally of vectorial form. A similar relationship has already been exploited to predict approximate structural profiles from sequences [7,8].

## Results and Discussion

### Vectorial Structure Profiles

To represent protein structural information in form of three-dimensional coordinates is highly redundant. Due to the stiffness of the polypeptide backbone, volume exclusion, and restrictions imposed by physical interactions between atoms, only a small subset of the combinatorially possible set of conformations is feasible for physical protein structures. This implies that a reduction of the mathematical description should be possible with minor loss of information.

A reduced representation of protein structure that is often used consists of the so-called contact matrix C_*ij*_, which is a binary matrix representing all amino acid pairs *i *and *j *whose distances lie below a cutoff distance *d*_th_. In the present work, contacts are defined based on minimum distances between heavy atoms (i.e. all but hydrogen) of amino acid pairs with a cutoff of *d*_th _= 4.5 Å. A contact matrix defined by the protein backbone, for instance by C_*α *_atom distances, would have the advantage that it is less dependent on the details of the side-chains and more conserved in evolution. Nevertheless, we prefer to adopt here a contact definition based on heavy atoms since this has a closer relationship to protein energetics and it yielded better results in our tests.

It was numerically shown in [9] that the contact matrix preserves the structural information of globular protein folds up to a level comparable with experimental resolution.

Several structural profiles associating each protein site *i *with a single real number *v*_*i *_can be derived from the contact matrix in a natural way. In [3] it was shown that one such representation, based on the principal eigenvector of the contact matrix, in the following denoted as PE, is sufficient to reconstruct the whole contact matrix for protein chains that consist of one structural domain, and consequently it is able to encode protein structure. Nevertheless, the PE is a meaningful structural profile only for single-domain protein chains. If more domains are present, or if a structure has relevant internal modularity below domain level, the contact matrix splits up into a number of modules that are highly intra-connected but only marginally inter-connected. In general, the principal eigenvector of such a matrix contains information about only the largest or most densely connected domain, while the remainders are distributed over the whole eigensystem.

An 'ad hoc' generalization of the PE, described in [10], is also applicable to multi-domain protein folds. This is achieved by assigning a small value to those components of the contact matrix whose corresponding residues are not in contact and were formerly set to zero. After computing the new principal eigenvector, a non-linear transformation is applied to recover the distribution of the original PE's components. This revised definition permits to consistently describe single- and multi-domain protein structures, keeping crucial properties of the original PE. In the following we call this profile the 'revised principal eigenvector' (revised PE).

Another more systematic way to extend the properties of the PE to modular protein structures consists of defining the 'generalized effective connectivity' profiles (GEC) [2] (UB, A.R.Ortíz, MP, FT: Effective Connectivity profile: A structural representation that  evidences the relationship between protein structures and sequences submitted), which are a family of structural profiles whose components *c*_*i *_self-consistently represent the effective contact density at site *i *in the native protein structure. The effective connectivity *c*_*i *_depends not only on the local contacts at site *i*, but it is a global property of protein structure. The principal eigenvector of the contact matrix is a specific member of this GEC family of profiles.

The relevance of the GEC structural profiles for the description of proteins derives from the fact that it is possible to naturally define a vectorial representation for protein sequences by associating the corresponding hydrophobicity value *h*(*A*_*i*_) to each amino acid *A*_*i*_. In the framework of folding models based on contact interactions, it is possible to show that the hydrophobicity profile (HP) associated with the optimally stable protein sequence belongs to the GEC family of the corresponding native structure [4]. This important property establishes a strong mathematical relationship between protein sequence and protein structure, both represented as vectors in the same space which, in turn, leads us to predict that the evolutionary average of the HP corresponding to stable protein sequences is very strongly correlated with some vector of the GEC family.

In UB, A.R.Ortíz, MP, FT: Effective Connectivity: A structural profile for multi-domain proteins, submitted, we proposed how to select a particular profile belonging to the GEC family in such a way that it depends only on protein structure and that it is strongly correlated with the average HP over a broad range of mean hydrophobicity values. In the following this profile will simply be called 'effective connectivity' (EC). For small single-domain protein structures without internal modularity, the EC essentially coincides with the PE.  In the same paper, it was also shown that the revised PE is related to a vector of the GEC family through a non-linear transformation, and that the revised PE and the EC are very strongly correlated, with correlation coefficient typically as large as 0.95.

Both the EC and the revised PE have been employed in the alignment framework discussed in this paper. We observed slightly better performance for the EC, which was therefore used as the standard for the alignment routine, whereas the revised PE is mentioned for some special cases where significantly different alignments are found using this profile. Nevertheless, the revised PE may be the better choice if fast computation of the profiles outbalances slightly higher accuracy.

The vectorial structure profiles revised PE and EC, together with contact maps and sequences can be freely downloaded from our web server at [11]. For more details please refer to the Methods section.

### Alignment Framework

In the framework discussed here, the task of finding a proper alignment of two protein structures is translated into the recognition of similar connectivity patterns in their corresponding structural profiles. This analogy is grounded on the assumption that the structural profile is conserved in protein evolution, like the overall topology of the protein structure that it describes.

In this way, we can use fast and simple comparison algorithms, while relevant non-local properties of protein structure are retained. Moreover, the resulting alignment is little dependent on spurious local similarities that could obliterate the recognition of far homologs. However, these local structural details have to be reintroduced in a second step, in order to obtain a more precise structural match.

Following this idea, we developed a structural alignment routine that consists of two steps. First, the alignment of the structural profiles is used to recognize global similarities. Second, a refinement step employs the atomic coordinates in order to improve the local structural superimposition.

#### Alignment Algorithm

**Figure 1:**
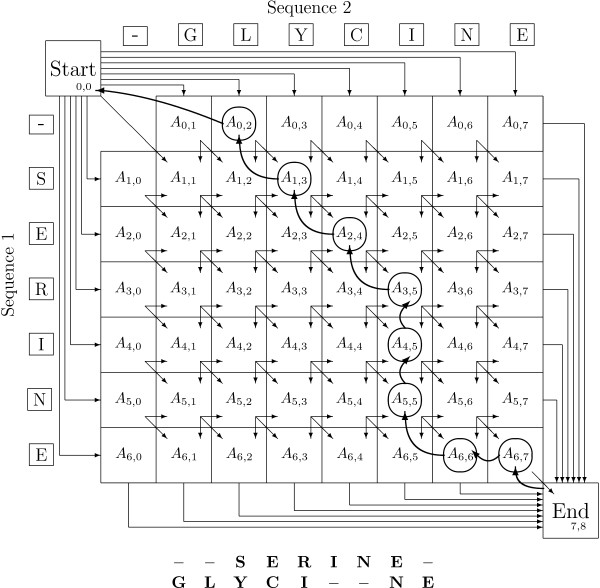
**Alignment Matrix**. The alignment matrix with a possible path encoding a specific alignment is depicted. Diagonal steps correspond to aligned amino acids while horizontal and vertical steps introduce gaps in chain 1 and 2, respectively. The path following arrows from 'End' to 'Start' refers to trace-back pointers set by Dijkstra's algorithm to mark the cheapest path found. Below the figure the alignment related to the marked path is displayed in gapped sequence description.

The profile alignment was designed similarly to 'traditional' sequence alignment routines like e.g. dot-matrix alignments. We represent every possible alignment of two proteins by a path through an alignment matrix *A*_*ij*_, as the one shown in Fig. 1. Possible alignments are defined as the line-up of two amino acid chains, together with an arbitrary number of inserted gaps of arbitrary length. Rare deviations from this pattern, like sequence repetition, mirrored parts, and replacement of groups of amino acids are not taken into account.

Building up this path, a diagonal step *A*_*i*-1, *j*-1 _→ *A*_*ij *_along the alignment matrix in Fig. 1 represents the alignment of amino acid $A_{i}^{\left(1\right)}$ from chain 1 with $A_{j}^{\left(2\right)}$ from chain 2. Horizontal and vertical steps introduce gaps in chain 1 and 2, respectively. The set of admissible paths consists of all combinations of steps starting in the upper left corner of the matrix, ending at the lower right.

The optimum alignment path minimizes the cost function described below, which depends on a set of parameters that are analogous to traditional 'substitution probabilities' for alignments and 'open/extend' penalties for gaps. However, in contrast to those, the penalties used here are directly dependent on the structures through their explicit dependence on profile components.

Evidently, the cost function must be such that the alignment of amino acids $A_{i}^{\left(1\right)}$ and $A_{j}^{\left(2\right)}$ is favoured if the associated profile components $c_{i}^{\left(1\right)}$ and $c_{j}^{\left(2\right)}$ are similar, so that the cost increases if aligned components are more different. Inserting a gap is penalized in two different ways. First, the chain in which the gap is inserted needs to be broken. From a structural point of view this is equivalent to a disruption of a number of contacts, which is less likely in parts of the chain that are more highly connected, since more contacts have to be broken. With a second penalty we model that it is less likely that the inserted chain part (that is opposite to the gap) is very highly connected to the rest of the structure because a higher number of contacts imposes stronger steric constraints.

The entangled use of these 'break' and 'insert' contributions to the gap penalty models the inherent ignorance of whether a gap in the alignment was caused by the deletion of a fragment from one chain during evolution, or by the insertion of a fragment in the other chain.

A detailed description of the path cost function and the parameters used can be found in the Methods section below.

Being equipped with a cost function, the cheapest path can be selected through very efficient standard algorithms. Here we used Dijkstra's shortest path algorithm [12], but dynamic programming algorithms, like Smith-Waterman [13] or Needleman-Wunsch [14] would have been equally applicable.

**Figure 2:**
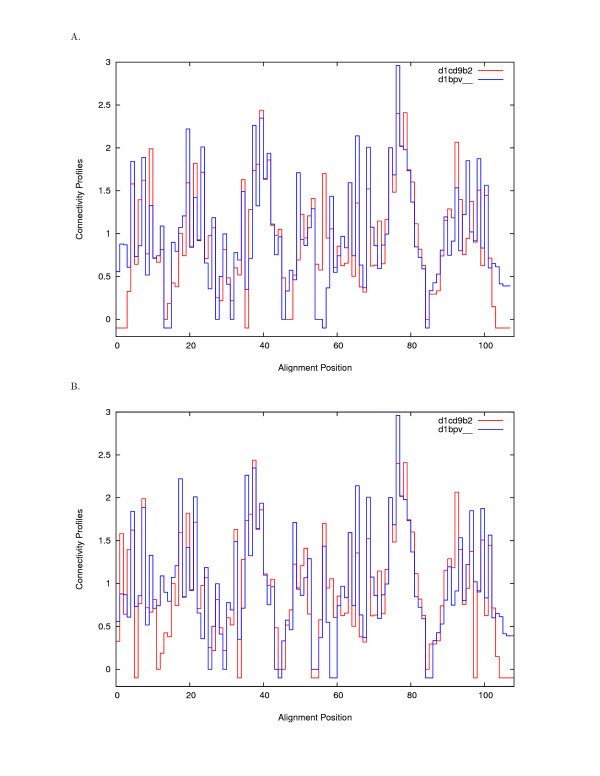
**Aligned Structural Profiles for Alignment Example 'd1cd9b2' vs. 'd1bpv__'**. Figure 2a shows the optimal profile alignment of the ASTRAL [33] domains 'd1cd9b2' and 'd1bpv__' as computed by the first step of SABERTOOTH without refinement. Gaps, as marked by small negative values, are inserted in regions with low connectivities, continuous patterns of larger connectivities are correctly aligned. Figure 2b shows the result for the same example as output by the full SABERTOOTH algorithm including refinement. Comparison to the result of the profile alignment shows only slight corrections introduced by the refinement routine while the overall similarity match is retained. The number of residues close in space after optimal rotation increases from 56 to 78 residues, PSI_profile _= 56.6% increases to PSI_refined _= 78.8%.  Improvement of the alignment is achieved by introducing obvious deviations from the overlap maximization of the structural profiles which implies that some local properties of the structures are not represented in the profiles, as expected by their construction. Three-dimensional superposition for this alignment is shown in Fig. 3.

The resulting optimal alignment can be represented in the form of superposed profiles, as in Fig. 2, upper plot. As expected, continuous regions of similar patterns are aligned and gaps are inserted in regions with low connectivities.

Up to this point our alignment algorithm does not use any other information besides of that encoded into the structural representation and the scoring function. The results presented below show that the algorithm is able to identify significant similarities in around 95% of the alignments of distant relatives from the test set discussed below.

We are aware that introducing other sources of information, such as secondary structure, prior domain decomposition, or biological knowledge of protein function, to name but a few, could further improve the alignments. However, this was deliberately omitted to keep the algorithm general, moving these issues to the structural representations and their possible future improvements.

In order to assess the alignment, we apply the standard MaxSub routine to the set of aligned residue pairs and compute the optimal rigid body rotation and translation that maximize the spatial superimposition of the two proteins, as described in [15] for MaxSub and in [16,17] for the rotation itself. This allows for the calculation of standard similarity scores based on coordinates and producing spatial views of the alignment.

#### Refinement on the Coordinates

As described above, through the MaxSub routine and the set of aligned residues we can derive the optimally superimposed set of coordinates, and from that we can compute the pairwise distances of all combinations of amino acids connecting the two protein chains. This detailed local information can then be exploited in a second alignment step in order to refine the alignment itself, similar in principle to other structural alignment algorithms.

For this goal, we firstly identify pairs of C_*α *_atoms that are closer in space than a threshold $d_{\text{th}}^{\text{refine}}$, disregarding whether these pairs belong to the set of aligned amino acids or not. Spurious pairs are sorted out by imposing the condition that only pairs that are member of sufficiently long continuous fragments of pairs *l *≥ *l*_min_are relevant. The values of these parameters must be chosen carefully since the result of the whole procedure is strongly influenced by their selection. By increasing the distance threshold and decreasing the minimum length parameter, more and more incidental pairs are selected. On the one hand, this results in a larger percentage of structural identity (PSI) for the refined alignment but, on the other hand, it lessens the significance of the alignment by interspersing it with spurious pairs that should actually not be included. Taking these factors into account we choose a minimum group length of four amino acids, *l*_min_= 4, and a distance threshold of $d_{\text{th}}^{\text{refine}}$ = 4 Å.

The set of amino acids effectively close in space is then used to restrict the possible paths through the alignment matrix, so that the second run of Dijkstra's optimization routine looks for the optimal path only around these identified groups of close pairs.

This second run of Dijkstra's algorithm incorporates close pair groups into the alignment where unambiguously assigned, it picks out the best choice in cases where more than one alternative is present, and it simply minimizes the path cost as before in areas that are not constrained.

Obviously, this kind of refinement is only able to improve the input alignment if the initial spatial superposition was already close to optimal. From comparison of the plots in Fig. 2, one can see that the refinement introduces only minor shifts into the alignment, keeping the originally assigned common core intact. Interestingly, when looking closer at the local details of the structures rather than comparing their global properties, one can see that the alignment can be improved by introducing obvious deviations from the optimal superimposition of the structural profiles. This implies that some local properties of the structures are not represented in the profiles, as expected by their construction.

#### Scoring the Alignment Results

After the refinement step, a second run of the MaxSub algorithm is used to obtain the optimal spatial superimposition through which we assess quality and significance of the final alignment. To this aim, we calculate the number of aligned residues and gaps, the number of residues close in space and the percentage, which is named percentage of structural identity (PSI), the coordinate RMSD (cRMSD), the sequence similarity of aligned residues, and the contact overlap. Furthermore, a *Z*-score measuring the statistical significance is computed from the PSI, as discussed below. Scores specific for profile alignments state the total optimized path cost, the summed cost of aligned regions, and the gap penalties.

**Figure 3:**
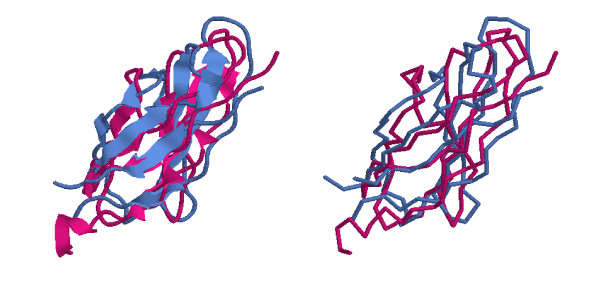
**Three-dimensional Superposition of 'd1cd9b2' vs. 'd1bpv__' after SABERTOOTH Alignment**. The alignment of 'd1cd9b2' vs. 'd1bpv__' as computed by SABERTOOTH including the refinement routine is shown in all-atoms form on the left hand side and in backbone form on the right hand side. The profiles corresponding to this alignment are shown in Fig. 2b.

In addition, different visualizations of the alignment result are computed: PDB-style coordinate files and a RASMOL [18] script file in C_*α *_or all-atoms shape, as well as plots of aligned profiles are included. Figure 3 shows the spatial superposition of an example alignment as computed by SABERTOOTH.

The program can be used freely at our web server [19]

#### Assessing the Significance of the Alignments

In order to distinguish biologically relevant relationships from superimpositions arising by chance, we assess the statistical significance of the alignments through the *Z*-score of the PSI, which measures the difference between observed and average PSI for optimal superimposition of unrelated pairs of structures, in units of standard deviation.

**Figure 4:**
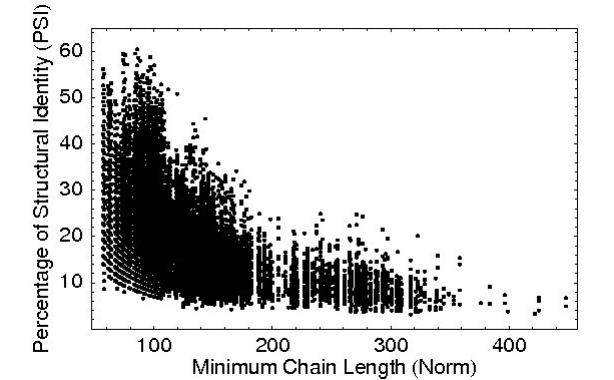
**Scatter Plot PSI over Norm for *Z*-score Determination**. The scatter plot shows the PSI over shorter chain length for the 31284 alignments of unrelated pairs in the *Z*-score set. Considerably high PSI values are found even for alignments with MAMMOTH and DaliLite *Z*-scores < 3.

**Figure 5:**
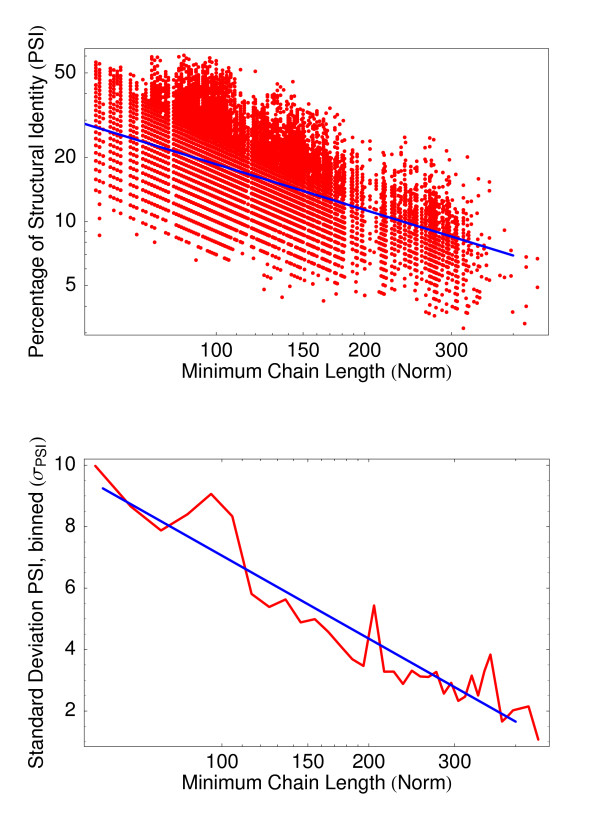
**Fits of Mean PSI and Standard Deviation for the *Z*-score**. The upper figure shows the log-log scatter plot of the PSI versus the length of the shorter chain for the *Z*-score set together with the power-law fit for the mean PSI that resulted in ⟨PSI⟩ = 494·min (*N*_1_, *N*_2_)^-0.712^. The lower plot shows the log-linear plot of binned PSIs' standard deviations. The exponential fit resulted in *σ*_PSI _= 25.00 - [3.896·log (min (*N*_1_,*N*_2_))].

Since the PSI of a pair of unrelated structures strongly depends on their lengths, we computed its mean and standard deviation as a function of the length of the shorter chain in each alignment using a set of 31284 alignments of pairs of non-related structures. The resulting PSI is represented in Fig. 4 versus length of the shorter chain. As expected from statistical reasoning, the plot shows that the PSI decreases for longer chain lengths, as described in [20,21]. This is rather intuitive since the same PSI corresponds to shorter aligned length for short chains, and it is more likely to get this aligned length by chance. The fitting of the parameters used in the *Z*-score computation is described in detail in the Methods section, the fits are shown in Fig. 5.

To verify that the *Z*-scores produced by our algorithm give an intrinsic and meaningful measure of the significance of the structural alignments, we tested whether they agree with those that result from other well-known algorithms (see Methods).

We performed this test by computing correlations between the *Z*-scores obtained through different algorithms for pairs of related proteins, finding correlation coefficients up to *cc*_1 _= 0.90 for SABERTOOTH vs. DaliLite and *cc*_2 _= 0.84 for SABERTOOTH vs. MAMMOTH. These high correlations show that the different algorithms are sensitive to the same 'signals' even though their individual accuracy is different. We show this pairwise correlation in Table 1. From this table, we furthermore learn that the correlation of all other tested algorithms with the algorithm CE is rather low, compared to the mutual correlations of these algorithms among each other. This seems to be a direct result of CE's peculiar behaviour, since the PSI obtained from CE alignments correlates rather weakly with those produced by all other algorithms assessed here.

**Table 1:** Correlation Coefficients for PSI and Z-score

	SABERT. (EC profile)	MAMMOTH	MAMMOTH mult	DaliLite	TM-align	CE	SHEBA
SABERTOOTH	1	0.79	0.84	0.86	0.85	0.61	0.81
MAMMOTH	0.84	1	0.85	0.84	0.81	0.64	0.80
MAMMOTH-mult	0.84	1.00	1	0.90	0.89	0.63	0.85
DaliLite	0.90	0.87	0.87	1	0.94	0.67	0.89
TM-align	0.53	0.59	0.59	0.62	1	0.66	0.88
CE	0.57	0.64	0.64	0.62	0.52	1	0.63

Correlation coefficients of rPSI values over the test set for the different algorithms are shown above the diagonal. The highest value of 0.94 between DaliLite and TM-align proves the very high level of agreement in the results. SABERTOOTH reaches 0.86 and 0.85 correlation with DaliLite and  TM-align, respectively.

The correlation of rPSI values translates to the *Z*-score as shown below the diagonal. MAMMOTH's *Z*-score obviously takes advantage of its more sophisticated *Z*-score fitting procedure using extreme value distribution methods to determine mean value and standard deviation of the PSI as functions of chain length.  SHEBA does not output comparable *Z*-scores.

For this analysis the *Z*-scores are obtained directly from the output of each program, in contrast to all other scores that are computed through our own assessment algorithm using the alignment merely as an input. This is necessary since the *Z*-score depends on the statistical properties of the optimal superimposition of unrelated pairs as computed through the very algorithm. Moreover, different programs can take care of the dependence on chain length in different ways. SHEBA does not output a  Z-score but from its high PSI correlation we expect comparable recognition behaviour in the sense of the above.

### Alignment Quality Assessment

**Figure 6:**
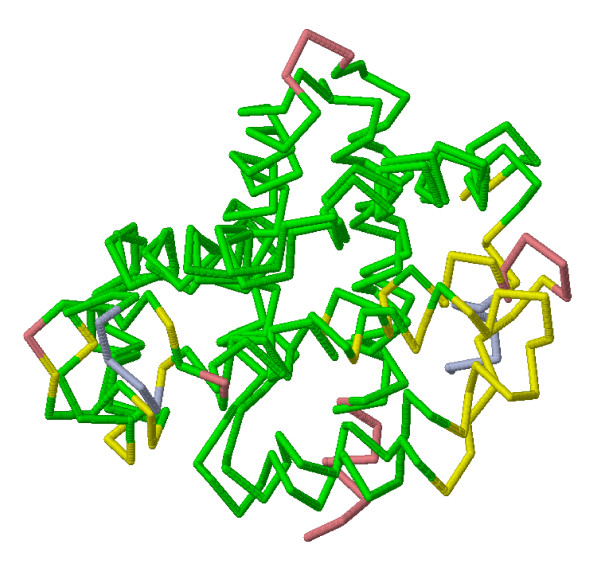
**Three-dimensional Superposition of 'd3sdha_' and 'd2gdm__' after SABERTOOTH Alignment; Colour-coded for Quality Assessment**. The alignment of 'd3sdha_' and 'd2gdm__' is shown after SABERTOOTH alignment and optimal rotation. Aligned regions that are closer in space than 4 Å are marked in green, aligned regions further apart are marked in yellow, and regions that are opposite to gaps in the alignment are marked in light blue and light red.

Since our algorithm compares protein structures using a representation that embodies the overall topology of a protein chain, it is expected to be largely tolerant in cases where local distortions tend to hide structural similarities. In fact, SABERTOOTH assigns PSI > 40% in nearly 95% of the alignments from the test set which consists of distant relatives that are related on superfamily level having less than 40% sequence identity. This proves a high recognition rate of the common core of distantly related proteins. The alignment is very accurate in the details as well. Figure 6 shows the superposition of the backbones of the structures with ASTRAL-IDs 'd3sdha___ _' and 'd2gdm__' after optimal rotation according to the SABERTOOTH alignment. A RASMOL script file for this example can be found in Additional File 1.

Aligned amino acid pairs that are closer in space than 4Å are coloured in green, marking the common core, whose size corresponds to the PSI, sections coloured in yellow are aligned but they are further in space than the PSI threshold. Light blue and light red chain sections are opposite to gaps. It can be seen that yellow parts point to local distortions that are correctly aligned, while gaps are introduced before the N- and after the C-terminus of the first chain and in loops of different lengths in the two chains.

With respect to the set of unrelated pairs used to fit the *Z*-score, SABERTOOTH assigns high PSI values to a number of alignments, even though the set consists of pairs from different folds that were checked to have MAMMOTH and DaliLite *Z*-score < 3.

Visual inspection of these alignments confirms that these high PSI values do not arise by chance but due to truly recognized similarities. In fact, the pairs with highest scores share a common module consisting of large portions of correctly aligned secondary structure elements, whereas tilted helices, twisted beta-sheets, and large insertions (or deletions) demand to assign them to different folds.

RASMOL script files for some examples from the test set can be found as supplementary material in Additional File 2.

Some specific problems of the algorithm are revealed by looking at alignments of related pairs from the test set that yield very small PSI values. The eight worst such cases consist of alignments between members of the SCOP superfamily 'Riboflavin synthase domain-like' (sf = 63380). These have barrel structure and are built up by anti-parallel beta sheets known also as the 'Greek key' motif. A rather complicated case from this superfamily is the chain with ASTRAL-ID 'd1ddga1'. Despite being considered as single-domain in the SCOP classification, it actually consists of two structural domains, the Greek key domain and an additional all-alpha domain, the latter built up from a number of loosely connected short helices. In some alignments with this structure, our algorithm fails to align the domains correctly, which seems to result partly from the structural representation used.

When comparing the results to those computed using the revised PE instead of the EC profile, some of the formerly problematic structures show very good results, i.e. the worst combination using the EC ('d1fdr_1' vs. 'd1ddga1') improves to PSI= 76% when using the revised PE as structural representation. But still, the abundance of anti-parallel beta structures in the problematic cases is obvious. Anti-parallel beta sheets are highly connected through hydrogen bonds of relatively short range along the sequence, a fact that is not explicitly considered in the profiles. This suggests that the structural representation used in the alignment routine might be improved through adequate incorporation of the hydrogen bonding network or secondary structure information.

Another negative influence on alignment performance could result from the symmetry in the structures of the above-mentioned superfamily that contain two opposing Greek key motifs. Alignment of structures with high symmetry leads to scoring functions with multiple solutions having very similar score.

### Alignment Quality Comparison

**Figure 7:**
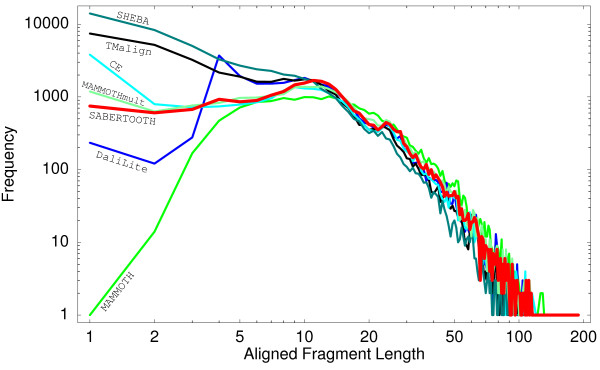
**Aligned Fragment Length Histograms**. Frequency of lengths of aligned fragments for alignments of the test set obtained with the algorithms assessed here. The behaviour found is very different for the different schemes. The total number of residues in fragments of sizes smaller than four sums up to 1.01% for SABERTOOTH, 0.13% for MAMMOTH, 1.18% for MAMMTOH-mult, 0.33% for DaliLite, 7.25% for TM-align, 2.22% for CE, and to 12.20% for SHEBA. Those aligned pairs are most likely not significant and should be omitted when the PSI is computed.

To evaluate the quality of SABERTOOTH's alignments it is necessary to compare it to competing algorithms. The best way to compare structural alignments is by visual inspection, but to make the comparison objective one has to use a quantitative score. In our opinion, the most expressive such score for a structural alignment is the PSI. As we mentioned above, when using the PSI one should take care that only truly significant superimpositions are counted in. Hence, PSI values have to be cleared from contributions from short spatial superimpositions, which may very likely arise by chance. Even if these matches are relevant, their expressiveness in this context is arguable and different tools deal very differently with this issue. Some algorithms apparently enhance the PSI to some extent by scattering tiny fragments of one to three amino acids along the alignments. This is quantified in Fig. 7, which shows the distribution of lengths of continuously aligned fragments produced by different algorithms.

The DaliLite algorithm shows a kink at length four in this distribution, with shorter fragments strongly penalized and also MAMMOTH attenuates short fragments. In contrast, the TMalign and the SHEBA algorithm over-represent these fragments.

To take this into account, we use a modification of the PSI throughout this paper that does not count in superimposed residues in fragments shorter than four continuous residues. We call this value the relevant PSI (rPSI). A hint that this treatment is reasonable follows from the comparison of sequence similarities with and without short fragments. Removing short fragments, sequence similarities increase for all algorithms which indicates that the mean sequence similarity of the removed fragments is smaller than for longer aligned fragments, suggesting that several of these short fragments are indeed spurious matches. A number of assessment scores, including sequence similarity based on the BloSum62 matrix, cRMSD, the number of aligned amino acids, and the number of aligned amino acids that are effectively close in space are listed in Table 2 for direct comparison of the algorithms.

**Table 2:** Various Scores for Comparison

	SABERT. (EC profile)	SABERT. (revised PE)	MAMMOTH	MAMMOTH mult	DaliLite	TM-align	CE	SHEBA
rPSI/%	67.8 ± 1.1	67.0 ± 1.1	51.3 ± 0.9	71.0 ± 1.2	73.6 ± 1.2	71.5 ± 1.2	64.2 ± 1.1	65.1 ± 1.1
PSI/%	68.2 ± 1.1	67.4 ± 1.1	51.3 ± 0.9	71.6 ± 1.2	73.7 ± 1.2	75.3 ± 1.3	64.6 ± 1.1	73.5 ± 1.2
cRMSD/Å	5.75 ± 0.10	5.84 ± 0.10	8.82 ± 0.15	5.63 ± 0.09	4.95 ± 0.08	2.85 ± 0.05	4.15 ± 0.07	5.12 ± 0.09
cRMSD/Å (core only)	1.90 ± 0.03	1.91 ± 0.03	1.91 ± 0.03	1.90 ± 0.03	1.86 ± 0.03	1.84 ± 0.03	1.91 ± 0.03	1.81 ± 0.03
aligned residues	395537	392528	410541	399948	392515	379196	340586	373857
contact overlap	57.3 ± 0.9	56.3 ± 0.9	44.7 ± 0.7	57.8 ± 1.0	61.0 ± 1.0	58.7 ± 1.0	50.3 ± 0.8	54.2 ± 0.9
rseqSim	8.0 ± 0.1	7.3 ± 0.1	-8.8 ± 0.1	6.4 ± 0.1	14.1 ± 0.2	15.4 ± 0.3	9. ± 0.1	15.8 ± 0.2
seqSim	8.1 ± 0.1	7.3 ± 0.1	-8.9 ± 0.1	6.0 ± 0.1	13.5 ± 0.2	11.2 ± 1.0	8.4 ± 0.1	9.4 ± 0.2
alignments rPSI< 40%	268/7.5%	262/7.3%	1014/28.4%	150/4.2%	18/0.5%	56/1.6%	483/13.5%	232/6.5%

Scores for the alignments of the different algorithms for quality assessment and comparison. All values are computed over the test set of 3566 alignments and are weighted by chain length. Shorter chain lengths from all alignments sum up to 434027 amino acids. Error values are shown under condition of statistically independent experiments.

The ranking of the algorithms agrees well over the different scores, even though they describe features on different levels of abstraction from the underlying proteins. Note that the rank correlation coefficient of rPSI with contact overlap is one while it is smaller than one for PSI with contact overlap. This constitutes another argument supporting that the treatment of small aligned fragments is reasonable.

**Figure 8:**
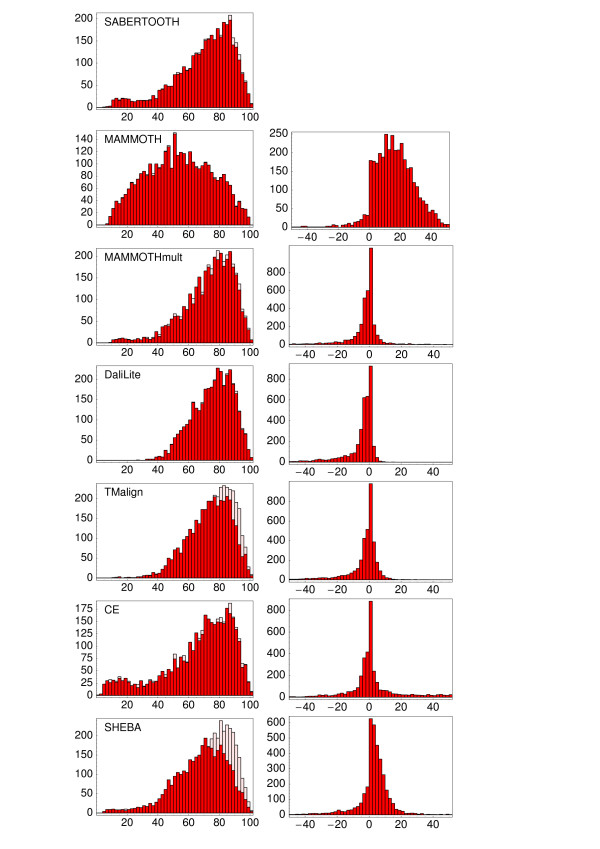
**rPSI Comparison Histograms, Differences to SABERTOOTH**. In the left column we report the histograms of the rPSI found by the different algorithms over the test set, the PSI (i.e. including short aligned fragments) are shown as shadows. The influence of short fragments is most dramatic for TM-align and SHEBA, as expected from Fig. 7. In the right column, differences to the results of SABERTOOTH are shown for direct comparison. Positive values connote higher rPSI for SABERTOOTH. Mean values may be found in Table 2.

In Fig. 8, the histograms of rPSI frequencies are plotted for the different algorithms compared, with the PSI displayed as shadows for the sake of completeness. Among the algorithms compared only DaliLite, MAMMOTH-mult, and TM-align perform better than SABERTOOTH. The ranking obtained is mainly consistent for the other quality measures, even though computed on different levels of abstraction and on different levels of the structural description. In particular, it is interesting to note that the sequence similarity score, which is based only on protein sequence, correlates very well with structural similarity scores when applied to the assessment of alignments of related proteins.

### Alignment Speed Comparison

To assess the computation speed of the different algorithms examined here, we measured the total time needed to compute all alignments in the test set. The fastest algorithm is the pairwise MAMMOTH. The slowest but most accurate competitor is DaliLite, which needs about 26.70 times as much CPU time as MAMMOTH. SABERTOOTH is only 4.23 times slower than MAMMOTH in the current implementation but it is nearly as accurate as DaliLite. In this sense, SABERTOOTH represents a good compromise between runtime and accuracy. A more detailed comparison is shown in Table 3, which lists CPU times needed for computing all of the 3566 alignment examples from the test set on an Intel(R) Xeon(TM) CPU 2.80 GHz. The third column shows computation times relative to the speed benchmark MAMMOTH.

**Table 3:** Computation Times for Comparison

Algorithm	CPU time/s	time rel.
SABERTOOTH	1726.3	4.23
MAMMOTH	408.4	1.00
MAMMOTH-mult	832.7	2.04
DaliLite	10902	26.70
TM-align	611.7	1.50
CE	2353	5.76
SHEBA	3533	8.65

Computation times for computing all of the 3566 alignment examples from the test set on an Intel(R) Xeon(TM) CPU 2.80 GHz are show as absolut values and relative to the speed benchmark MAMMOTH (pairwise).

### Detailed Comparison of Established Algorithms

#### MAMMOTH and MAMMOTH-mult

The MAMMOTH program [22] is the fastest algorithm assessed here. As well as the SABERTOOTH algorithm, it is based on the idea of aligning structural profiles that represent the proteins to be aligned, but in the case of MAMMOTH these profiles are obtained through local information on the dihedral angles between residues consecutive in the protein chain. In this sense, it may be viewed as complementary to our approach, which uses global profiles.

MAMMOTH was initially developed for comparison of large numbers of theoretical models, making computational time the prime duty. In this sense it may be viewed as a maximum speed benchmark. Due to its high speed, MAMMOTH is applicable to very large sets of alignments as those needed for database clustering. In contrast, its recognition rate is low. It is similar to that of SABERTOOTH's profile alignment routine without refinement (data not shown), which indicates that the quality of MAMMOTH alignments can be improved through a similar post-processing when higher accuracy is needed.

This has indeed been done for the multiple alignment program MAMMOTH-mult [23]. It carries out all-vs-all alignments using the pairwise MAMMOTH routine to construct a guiding tree on the basis of which the multiple structural alignment is deduced. Following this tree, new structures are aligned to the groups already joined, ordered by decreasing similarity. At this stage, after the profile based alignment, the program applies a post-processing routine based on a scoring function that depends on pairwise inter-atomic distances calculated after optimal superimposition. This post-processing routine is able to improve MAMMOTH's performance to a level close to DaliLite while its computation times grow by slightly more than a factor of two. This makes MAMMOTH-mult a very good choice even for pairwise structural alignment, and prompts at the convenience to include a similar post-processing step also in its pairwise version.

#### DaliLite

The DaliLite algorithm [24] uses a structural representation based on the distance matrices of the C_*α *_trace, which results in a high ability to recognize structural similarities and in a high precision in the details. Unfortunately, the algorithm is slow so that its applicability is very restricted.

Since DaliLite does not output any alignment when it does not find significant similarity, the pairs for which this happened were removed from the test set, although this may favour DaliLite in comparison to the other programs. Surprisingly, in 31 out of 36 of the cases in question (data not shown) SABERTOOTH finds PSI > 40% and in 14 cases even PSI > 75%. This behaviour could be due to a systematic weakness of the DaliLite algorithm, which is a bit too strict when trying to sort out alignments of unrelated structures to improve its speed on large data sets.

Besides of this, DaliLite assigns significant PSI values in nearly all alignments of the test set which makes it  first choice for an accuracy benchmark.

#### TM-align

The TM-align algorithm [25], at first glance, shows even higher accuracy than DaliLite in combination with a speed that is only 1.5 times slower than MAMMOTH. But if one looks closer at the results one finds that the high PSI results from a very high number of aligned fragments of short length. For TM-align more than 7% of its aligned residues are member of fragments shorter than four amino acids. This is more than seven times more than for SABERTOOTH. Looking at the rPSI distribution instead, TM-align's recognition rate is slightly worse than DaliLite's and the mean value is only 3.7% higher than that of SABERTOOTH.

#### CE

The CE algorithm [26] seems to over-represent short fragments of length one, but not longer ones. In spite of that, PSI and rPSI values found are nearly identical. There is a high number of alignments for which the CE algorithm does not recognize significant similarity, visible in Fig. 8 as a hump of small rPSI values that contains more than 13% of the test set. This difficulty in recognizing distant similarity is also evident from the fact that PSI (and *Z*-score) values obtained with the CE algorithm are significantly less correlated with those of the other algorithms than those are between each other, as shown in Table 1.

#### SHEBA

The SHEBA algorithm [27], like TM-align, shows high PSI values that drop by more than 8% in the mean when looking at the rPSI. This results from the extremely high number of more than 12% of aligned amino acid pairs in fragments below four amino acids in length. Besides of this, SHEBA shows good agreement with the other algorithms.

## Conclusion

We present a new approach to protein structural alignments. We could show that structure representations in form of vectorial profiles based on the global topology of protein structure allow structural alignments of a quality comparable to other state-of-the-art methods. The SABERTOOTH structural alignment server implements this alignment framework. It performs comparably to algorithms based on coordinate derived descriptions and represents a good compromise between alignment accuracy and computational speed. We could show this by statistical analyses and comparison to established competitors which we use as objective references.

One of the strengths of our alignment scheme is its generality, since it can be applied to different vectorial structure representations and, hence, allows for incorporation of more elaborated structural profiles developed in the future. Even more interesting is the possibility to utilize structural profiles predicted from sequences in future uses of the framework. Doing so, all flavours of protein alignments, i.e. structure-to-structure, sequence-to-sequence, and sequence-to-structure, can be carried out within the same scheme. Furthermore, extension to multiple alignments using mixed sets of sequence and structure profiles is possible.

We are positive that when doing so our approach can help to better exploit available protein structure and sequence data by allowing for analyses of correlations between different vectorial structure representations derived from structure on the one hand and from sequences on the other hand. This might assist in gaining deeper insight into protein folding.

## Methods

### Structural Profiles

#### Principal Eigenvector of the Contact Matrix

The contact matrix *C*_*ij *_of a protein structure is a binary symmetric matrix with elements *C*_*ij *_= 1 if amino acids at positions *i *and *j *are in contact and *C*_*ij *_= 0, otherwise [9]. Two residues are defined to be in contact if at least one pair of heavy atoms, one belonging to each amino acid, are less than 4.5Å apart.

Other contact definitions, e.g. based on C_*α *_distances, are possible [9]. Additionally, only residues separated by at least three positions along the chain are considered to be in contact, i.e. *C*_*ij *_= 0 if |*i *- *j*| < 3, to ignore trivial short range contacts.

Since *C*_*ij *_is a symmetric matrix, all its eigenvalues are real and it has a complete orthonormal set of real eigenvectors. The principal eigenvector *c*_*i *_(PE), i.e. the eigenvector associated with the largest eigenvalue, is a structural profile that embodies the most relevant properties of protein structure. Its components are all of the same sign, which we chose to be positive. The PE maximizes the quadratic form ∑_*ij *_*C*_*ij *_*c*_*i*_*c*_*j *_under the normalization constraint $\sum_{i}c_{i}^{2}=1$. In this sense, *c*_*i *_can be interpreted as the effective connectivity of the amino acid at position *i *embedded in the structure, since positions with large *c*_*i *_are in contact with as many as possible positions *j *with large *c*_*j*_.

As shown in [3] the PE contains sufficient information to reconstruct its contact matrix and, hence, describes an equivalent representation of protein structure itself. But its applicability is restricted to single-domain protein structures with low internal modularity because the contact matrix of multi-domain structures splits up into a number of modules and the structural information is distributed over the whole eigensystem. In general, the principal eigenvector of such a matrix describes the most densely packed domain, only.

#### Revised Principal Eigenvector

An 'ad hoc' generalization of the principal eigenvector, introduced in [10], extends the applicability of structural profiles to multi-domain protein folds. This is achieved by modifying the contact matrix so that pairs of residues not in contact are assigned the small value *ε*(*N*) = min{*ε*_max_, *ε*_0_/(log *N *- *ε*_1_)} with *ε*_max _= 0.01, *ε*_0 _= 0.02, and *ε*_1 _= 2, instead of zero. The principal eigenvector of this revised contact matrix ${\tilde{C}}_{ij}$ is subsequently transformed in such a way that the transformed components show the same distribution as those of the original PE. The empirically derived parameters are adjusted to be just large enough to connect the different modules of a contact matrix ${\tilde{C}}_{ij}$, resulting in non-zero values in nearly all components of the revised principal eigenvector ${\tilde{c}}_{i}$.

This revised definition permits consistent description of single- and multi-domain protein structures, keeping crucial properties of the original definition.

#### Effective Connectivity Profile

Another approach to define a vectorial representation for single- and multi-domain protein structures is described in [2] (UB, A.R.Ortíz, MP, FT: Effective Connectivity: A structural profile for multi-domain proteins, submitted). This more general definition includes the PE, the revised PE [10], and structural profiles derived from protein sequences into a family of profiles called the 'generalized effective connectivity' family (GEC).

All members of the GEC family share the properties that (a) they maximize the quadratic form Q = ∑_*ij *_*C*_*ij*_*c*_*i*_*c*_*j*_, (b) their mean value is fixed to ⟨*c*⟩ = 1 to choose a normalization of the GEC components, (c) their mean square component is fixed to ⟨*c*^2^⟩ = *B *> 1.

For the special choice of $B=B_{\text{cont}}=\frac{\langle {\text{cont}}_{i}^{2}\rangle }{\langle {\text{cont}}_{i}^{2}\rangle }$ with cont_*i *_= ∑_*j *_*C*_*ij*_, the contact vector, we obtain the effective connectivity profile EC that is used in this paper. This profile can be expressed as a weighted sum of the vectors of the eigensystem of the contact matrix *C*_*ij*_, with weights gradually introducing contributions from more eigenvectors from *C*_*ij*_'s eigensystem when structures described get more modular.

It was also shown that the EC is nearly identical to the PE for small single-domain structures with low internal modularity, and that for all structures it is highly correlated with the revised PE. The significant correlation between the hydrophobicity profile of the protein sequence HP and the PE, valid for small single-domain proteins, is translated to multi-domain structures when using EC or revised PE profiles.

### Set of Structures Used

For training and testing the algorithm and for fitting the *Z*-score, we used a set of structures derived from the '29SCOPsf' set described in more detail in [29]. The set consists of 525 structures from 29 SCOP [30] superfamilies (release 1.69) that constitute a representative collection of common structural motifs. All superfamilies are from different folds of the SCOP classification and cover the four major SCOP classes all alpha, all beta, alpha+beta, and alpha/beta.

### Cost Function

The penalties that build up the path cost function are divided into three terms.

1. Aligned components of the structural profiles, corresponding to position *i *in the first profile and position *j *in the second profile, are penalized by a term that grows with their absolute difference,

$$M_{ij}={\left|c_{i}^{\left(i\right)}-c_{j}^{\left(2\right)}\right|}_{}^{p_{\text{align},\exp}}$$

with *p*_align,exp _as a tuneable parameter that will be referred to as scaling exponent.

2. Breaking chain *s *between residue *i *and *i *+ 1 is penalized by

$$B_{i}^{\left(s\right)}=p_{\text{break},\text{fac}}\cdot {\left(\frac{c_{i}^{\left(s\right)}+c_{i+1}^{\left(s\right)}}{2}\right)}^{p_{\text{break},\exp}}$$

with parameters *p*_break,fac _and *p*_break,exp_. This is based on the expectation that it is less likely to break a chain at a strongly constrained position with large components *c*_*i *_and *c*_*i*+1_.

3. An insertion of length *n*_*j *_in chain s at position *j *+ 1 opposite to a gap in the other chain, consisting of the components $\left[c_{j+1}^{\left(s\right)}\cdots c_{j+n}^{\left(s\right)}\right]$ is penalized by

$$I_{j}^{\left(s\right)}=p_{\text{insert},\text{fac}}\cdot \sum_{k=j+1}^{j+n}c_{k}^{{\left(s\right)}^{p_{\text{insert},\exp}}}$$

with parameters *p*_insert,fac _and *p*_insert,exp _and with s = {1, 2} selecting the chain. This is based on the expectation that strongly connected residues are less likely to form part of an insertion, or to be deleted from the other chain.

As a result, the complete path cost function is given by

$$\begin{array}{lll} S & = & \sum_{\forall (i,j)\in \text{P}}M_{ij}+\\ & & \sum_{\forall i\in {ℬ}^{\left(1\right)}}B_{i}^{\left(1\right)}+\sum_{\forall i\in {ℬ}^{\left(2\right)}}B_{i}^{\left(2\right)}+\\ & & \sum_{\forall j\in {ℐ}^{\left(1\right)}}I_{j,n_{j}}^{\left(1\right)}+\sum_{\forall j\in {ℐ}^{\left(2\right)}}I_{j,n_{j}}^{\left(2\right)} \end{array}$$

where P is the set of all aligned pairs of amino acids, ${ℬ}^{\left(s\right)}$ the set of all positions *i *after which chain *s *is broken, and ${ℐ}^{\left(s\right)}$ the set of all insertions of length *n *after position *j *in chain *s*.

For our implementation we found that the set of parameters displayed in Table 4 that scale and weight the different effects is close to optimal. The scaling exponents were found to be valid within structures and at their termini. Different values were found for the weighting factors for chain insertion, as expected from sequence alignment parameter values, even though the difference of the parameters is much smaller than for sequence alignments. An additional parameter for weighting the break of the chain at a terminus, i.e. to assign gaps before or after the chain, can trivially be omitted.

**Table 4:** SABERTOOTH Alignment Parameters

Parameter	EC	revised PE
*p*_align,exp_	1.60	1.79
*p*_break,fac_	1.79	3.25
*p*_break,exp_	1.57	0.38
*p*_break,term,exp_		
*p*_insert,fac_	0.43	0.83
*p*_insert,term,fac_	0.31	0.58
*p*_insert,exp_ *p*_insert,term,exp_	1.77	0.89

Penalties for alignment of positions, breaking a protein chain and for inserting a chain fragment opposite to a gap. The same parameter values are used in the profile alignment as well as in the refinement routine. Please refer to UB, A.R.Ortíz, MP, FT: Effective Connectivity: A structural profile for multi-domain proteins, submitted, for the definition of the EC profile and to [10] for the revised PE.

### Parameter Training

As any automatic alignment procedure, the algorithm described depends on adequate selection of its six parameters for opening and extending gaps. To obtain an optimal set of parameters, a Monte Carlo scheme was conducted starting from a manually adjusted parameter set. The decision whether to accept a preliminary parameter change or not was based on the value of the sum of residues close in space after optimal rotation, not using the refinement routine.

The training set used is a randomly selected subset of the pairs of chains from the 29SCOPsf set that are assigned to the same superfamily. Alignments with a DaliLite [24] or MAMMOTH [22]*Z*-scores smaller than four were removed from the training set, to ensure that it only contains pairs for which a well defined result exists. The training set consists of 235 alignments.

### Alignment Quality Assessment Routine

An automatic routine was set up to assess the quality of the alignments produced by our algorithm, as well as of alternative ones produced by well established programs. Alignments were computed for the 3566 pairs of structures from the same SCOP superfamily in our dataset not used in the training set, so that test and training sets are disjoint.

For all examples, the results of the different algorithms were inserted into our own procedure for computing the optimal rotation using MaxSub and the scores for quality assessment that are listed in Table 2. To make sure that the alignments of the different algorithms are comparable, their output was processed in order to agree in amino acid sequence. This was mandatory, due to different behaviours of the algorithms in reading PDB files and displaying results.

### Scores Computed for Quality Assessment

To allow for objective quality assessment and comparison of the algorithms used here, the following scores were computed.

The percentage of structural similarity (PSI) counts the number of aligned amino acid pairs whose C_*α *_atoms that are closer in space then 4 Å after optimal rotation normalized by the length of the shorter chain in the alignment. rPSI discards fragments shorter than four aligned amino acids from this value.

The root-mean-square deviation of the coordinates (cRMSD) is computed over all aligned pairs or, in the 'core only' case, over those pairs only that contribute to the PSI.

The contact overlap is deifned as

$$Q=100\cdot \frac{\sum_{ij}C_{ij}{C^{\prime }}_{ij}}{\min\left(\sum_{ij}C_{ij},\sum_{ij}{C^{\prime }}_{ij}\right)}$$

Where *C*_*ij *_and ${C^{\prime }}_{ij}$ are the aligned contact matrices of the structures compared, obtained using the minimum distance between heavy atoms with a threshold *d*_th _= 4.5 Å.

The sequence similarity is computed using the BloSum62 similarity matrix and is normalized to a percentage. rSeqSim again discards aligned residues in short fragments.

The alignment test set is available as Additional File 3.

### *Z*-score Fitting

The *Z*-score is generally defined as

$$Z=\frac{\text{PSI}-\langle \text{PSI}\rangle }{\sigma_{\text{PSI}}}.$$

To determine the quantities ⟨PSI⟩ and *σ*_PSI _needed to compute the *Z*-score we aligned 31284 pairs of unrelated structures. We used the same set of structures introduced above, but this time we aligned structures from different SCOP superfamilies where pairs with DaliLite or Mammoth *Z*-score larger than 3 were sorted out to make sure that the structures in the sample alignments are reasonably unrelated. The values for ⟨PSI⟩ and σ_PSI _were obtained by fitting functions to the data shown in Fig. 4.

While the mean follows a power-law decay

⟨PSI⟩ = *a*·min (*N*_1 _, *N*_2_)^*b*^,

the standard deviation is better matched by an exponential function

*σ*_PSI _= *c *- [*d*·log (min (*N*_1_, *N*_2_))].

The fits resulted to

⟨PSI⟩ = 494·min (*N*_1 _, *N*_2_)^-0.712^

and

*σ*_PSI _= 25.00 - [3.896·log (min(*N*_1_, *N*_2_))].

as shown in Fig. 5.

### Computational Complexity

The computational complexity of the procedure is composed of that of Dijkstra's shortest path algorithm [12] and the MaxSub routine.

We implemented the Dijkstra algorithm using a Fibonacci heap to find the cheapest vertex in each step. In this case, the algorithm has complexity O(|*E*| + |*V*| log |*V*|), where |*V*| is the number of vertices and |*E*| is the number of edges, i.e. the elements and arrows in the alignment matrix. For the initial profile alignment |*V*| = (*N*_1 _+ 1)·(*N*_2 _+ 1) + 1 with *N*_1 _and *N*_2 _as the number of amino acids in the protein chains, taking also into account the additional first row and column that permit leading gaps.

From this we find

|*E*| = (*N*_1 _+ *N*_2 _+ 1) + 2(*N*_1 _- 1 + *N*_2 _- 1) + 3((*N*_1 _- 1)(*N*_2 _- 1)) + (*N*_1 _+ *N*_2 _+ 1) + 2((*N*_1 _- 1)(*N*_2 _- 1))

for edges leaving the 'start' element,  leaving first row and column elements,  from inner matrix elements,  connected to 'end', and  additional double steps needed to implement the break/insert behaviour.

The complexity of the MaxSub algorithm is $O(N_{\text{MaxSub}}^{2})$, where *N*_MaxSub _≤ min (*N*_1_, *N*_2_) is the number of aligned amino acid pairs.

For the second run of Dijkstra's algorithm the refinement routine the restrictions imposed on the possible paths largely reduce the number of edges in the alignment matrix. This reduces the combined run time of Dijkstra's algorithm together with MaxSub in the second run to approximately 20% of that of the first run, over the test set discussed above.

The overall complexity is

$$O_{\forall }=O(N_{\text{Dijkstra}}\cdot \log N_{\text{Dijkstra}})+O(N_{\text{MaxSub}}^{2}),$$

with *N*_Dijkstra _= *N*_1_·*N*_2_. Both algorithms can be implemented very efficiently and allow for very short computation times, as discussed below.

### Implementation

The current program was implemented in C++ making extensive use of object-oriented design principles. Data representations and algorithms from the Standard Template Library and the Boost C++ libraries [31] were utilized. Some routines from LAPACK [32] were employed as well. The resulting code is presently optimized to allow for flexible modification rather than computational speed.

### Other Algorithms used for Comparison

Six different alignment algorithms that are freely available on the Internet have been used as references for comparison.

The **MAMMOTH **algorithm [22] was selected as speed reference due to its very short computation times. It is based on the idea of aligning structural profiles that are derived from the dihedral angles between consecutive residues in the protein backbone.

The **MAMMOTH**-**mult **algorithm [23] uses the same alignment routine as the pairwise version but then it applies an additional post-processing step, similar in spirit to the one implemented here.

The **DaliLite **algorithm [24] aligns C_*α *_contact matrices in a heuristic scheme of combining overlapping submatrices to get the final result. This makes the algorithm the slowest of our references but the precise coordinate representation results in the highest accuracy found here. We consequently use DaliLite as the accuracy benchmark.

The **TM-align **algorithm [25] achieves high alignment quality in short computation times by performing a two step algorithm. An initial alignment is computed mainly based on secondary structure information which is followed by a heuristic iteration scheme.

The **CE **algorithm [26] performs combinatorial extension of formerly defined aligned fragments based on local geometry in the protein structures compared.

The **SHEBA **algorithm [27] utilizes so-called 'environmental profiles', containing primary, secondary, and tertiary structure information for an initial alignment which is then iteratively refined by analysing the superimposed C_*α*_-traces.

## Availability and Requirements

Project name: SABERTOOTH Alignment Server

Project home page: 

Licence: The web services provided may be freely used without any charge.

## Authors' contributions

MP and UB designed research; FT and MP developed the alignment algorithm; FT coded and tested the alignment algorithm; FT, UB, and MP analyzed results; FT, UB, and MP wrote the paper. All authors read and approved the final manuscript.

## Supplementary Material

Additional file 1 RASMOL Script File for Alignment Example Fig. 6. The RASMOL script file 'd3sdha__d2gdm__maxSub.tcl' is the example shown in Fig. 6 in this publication. It can be viewed with RASMOL [18], freely available on the Internet. Click here for file

Additional file 2 RASMOL Script Files. The file contains RASMOL script files '1-d1bhe__d1daba__maxSub.tcl', '2-d1cd9b2 d1bpv maxSub.tcl', and '3-d1gca__d1dbqa__maxSub.tcl' for alignments of structures from the test set in quality assessment colour coding. The files '4-d1k0ma2 d1de4c3 maxSub.tcl', '5-d1gnwa2_d1dp4a__maxSub.tcl', and '6-d1iyha2_d1cg2a1_maxSub.tcl' contain RASMOL script files in all atoms depiction with alignments of structures from different SCOP folds but from the same secondary structure classes. Click here for file

Additional file 3 Alignment Test Set. The file 'alignment_testset.dat' contains the test set used for quality assessment. Two columns listing ASTRAL-IDs of the 3566 alignments as described above. Click here for file
